# Experimental Evaluation of Anticancer Efficiency and Acute Toxicity of Anthrafuran for Oral Administration †

**DOI:** 10.3390/ph13050081

**Published:** 2020-04-28

**Authors:** Andrey E. Shchekotikhin, Helen M. Treshalina, Michael I. Treshchalin, Eleonora R. Pereverzeva, Helen B. Isakova, Alexander S. Tikhomirov

**Affiliations:** 1 Gause Institute of New Antibiotics, 11 B. Pirogovskaya Street, Moscow 119021, Russia; funky@beatween.ru (M.I.T.); pereverzeva-ella@yandex.ru (E.R.P.); ebisakova@yandex.ru (H.B.I.); tikhomirov.chem@gmail.com (A.S.T.); 2 Federal State Budgetary Institution «National Medical Research Center of Oncology of N.N.Blokhin», Ministry of Health of Russia, 24 Kashirskoye sh., Moscow 115548, Russia; treshalina@yandex.ru

**Keywords:** anthrafuran, antitumor activity, oral administration, acute toxicity, *LD*_50_

## Abstract

The new antitumor agent anthrafuran has demonstrated a consistent effect in murine tumor models when administered parenterally due to the simultaneous inhibition of multiple cellular targets such as topoisomerases I/II and protein kinases. In this study, we assessed the anticancer efficiency and acute toxicity of anthrafuran administered orally. The action of anthrafuran was studied on transplanted tumor models which included P388 leukemia, Ca755 mammary adenocarcinoma, LLC lung carcinoma, and T47D human breast cancer xenografts on Balb/c nude mice. A significant antitumor efficacy of oral anthrafuran was revealed for all tested tumor models as follows: T/C_max_ = 219% for P388, TGI_max_ = 91% for Ca755, TGI_max_ = 84% with CR_max_ = 54% for LLC, and T/C = 38% for T47D. The optimal treatment schedule of orally administered anthrafuran was 70–100 mg/kg given daily for five days. The *LD*_50_ value of orally administered anthrafuran (306.7 mg/kg) in mice was six times higher than that for i.p. administration (52.5 mg/kg). The rates of antitumor efficacy and acute toxicity indicate the high potential for further research on anthrafuran as a new original oral anticancer multitarget agent with an expected satisfactory tolerability and bioavailability.

## 1. Introduction

Most anticancer drugs are administered parenterally because this route provides fast and maximal bioavailability, and therefore accurate dosing during the entire course of chemotherapy [1,2]. However, parenteral administration has some adverse effects because it typically requires hospitalization, nursing, and palliative treatment. Moreover, intravenous (i.v.) chemotherapy regimens are generally designed to deliver the maximal tolerable dose of cytotoxic agent, which may lead to hazardous side effects on normal tissues. These limitations have shifted the focus in cancer chemotherapy from the i.v. administration to oral therapy, which can be carried out by self-administration [3,4]. Currently, approximately 20–30% of anticancer drugs have formulations for oral administration, and their market share is growing rapidly [5]. However, as a rule, the efficacy of most anticancer drugs for oral administration is frequently limited due to reduced bioavailability, insufficient water solubility, or poor metabolic and pharmacokinetics characteristics [6,7,8,9]. Therefore, novel orally administered agents must have better formulation to achieve adequate bioavailability. Thereby, the search for novel orally bioavailable agents and the creation of orally delivered formulations for already marketed medications are the current directions in anticancer drug development [10].

**Figure 1:**
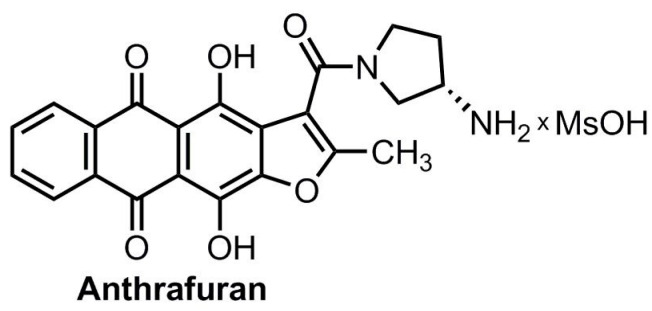
Anthrafuran structure.

Heterocyclic derivatives of anthracenedione efficiently inhibit tumor cell growth demonstrating advantages over prototype anthracyclines [11]. These compounds interact with several intracellular targets involved in cancer progression including topoisomerases, telomerase, protein kinases, and G-quadruplex structures of nucleic acids [12]. Among the newly synthesized derivatives we identified (*S*)-3-(3-aminopyrrolidine-1-carbonyl)-4,11-dihydroxy-2-methylanthra[2,3-*b*]furan-5,10-dione (anthrafuran, Figure 1) as the hit compound with submicromolar potency for several tumor cell lines [13,14]. Importantly, anthrafuran is also efficient against tumor cells with the molecular determinants of altered drug response such as the multidrug resistance (MDR) transporter P-glycoprotein (Pgp) or inactivation of p53. Anthrafuran induced apoptosis via inhibition of topoisomerases I/II, Aurora B protein kinase, and generation of oxidative stress [13,14]. The anticancer potency of **1** administrated intraperitoneally (i.p.) was supported by a pronounced therapeutic effect in the murine models of transplanted P388 lympholeukemia and its MDR-variant P388/DOX, as well as B16/F10 melanoma [15].

It is well known that classical cytotoxic agents, including antitumor anthraquinone derivatives (e.g., doxorubicin, rubomycin, and mitoxantrone), are administered parenterally owing to their instability or low absorption from the gastrointestinal tract [16]. However, the semi-synthetic anthracycline idarubicin demonstrates a high antitumor efficiency and reduced side toxic effects upon oral (p.o.) administration [17]. High chemical stability, relatively high lipophilicity, and a low efflux of anthrafuran by Pgp [13], which is expressed in the intestinal epithelium, can result in an acceptable absorption from the gastrointestinal tract. Comparative preliminary pharmacokinetic and the acute toxicity studies for various routes of administration in rats showed that anthrafuran had a good bioavailability upon p.o. intake and a lower general toxicity than after the parenteral administration [18]. Evaluation of the chronic toxicity and neurotoxic properties in rats also showed that animals well tolerated oral treatment with anthrafuran [19,20]. The side effects (e.g., inhibition of the motor and exploratory activity) were reversible within 30 days. This study presents the results of a preclinical investigation of the antitumor efficacy in vivo on leukemia and solid tumor models, as well as the acute toxicity of anthrafuran after oral administration in mice, while all previous pharmacokinetic and toxicological studies [18,19,20] have been performed in rats.

## 2. Results and Discussions

### 2.1. Antitumor Efficacy

The parenteral administration of anthrafuran showed the highest efficacy in leukemia models [13,15]. Thus, the investigation of oral administration was started with i.p. transplanted P388 leukemia. Table 1 shows that the range of tolerated daily doses in the course of a five-day treatment was 40–100 mg/kg (total effective dose (ED_Σ_) was 200–500 mg/kg). We observed a dose dependent antitumor efficacy, with a significant treatment-to-control (T/C) ratio. The autopsy showed that the sizes of lymph nodes or ascites in treated groups were the same or smaller than the respective parameters in the control cohorts. Anthrafuran at a dose of up to 100 mg/kg was well tolerated without any side effects or toxicity related death. However, the maximum tested single dose of 120 mg/kg was toxic for the majority of animals in the treatment group and could only be administrated twice or three times daily.

Comparison of the time course revealed that a five day administration was optimal; 80 mg/kg daily (total 400 mg/kg) resulted in the greatest T/C value of 190% independently of the interval between the application of anthrafuran (24 h or 48 h, Table 2). A longer treatment provided no benefits (Table 2).

**Figure 2:**
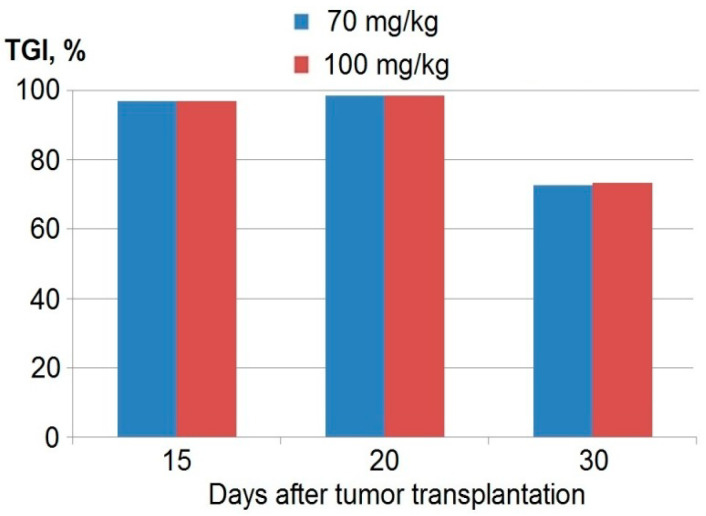
Tumor growth inhibition (TGI) of Ca755 mammary adenocarcinoma after oral anthrafuran treatment for 5 days.

The oral administration of anthrafuran with a single dose of 70 or 100 mg/kg daily for five days, starting from the third day after tumor transplantation, revealed a significant tumor growth inhibition (TGI) of Ca755 mammary adenocarcinoma with TGI_max_ = 91% (*p* < 0.05). The significant antitumor effect of anthrafuran was observed during 30 d after tumor transplantation without significant differences for both treated groups (Figure 2).

The five-day schedule for the oral administration of anthrafuran was used in the next stage of the efficacy study on solid tumors models. It has been shown that in mice with subcutaneously inoculated LLC, the selected mode of treatment (5 × 80 mg/kg for 2–6 d after tumor transplantation) led to a significant suppression of tumor growth. Seven out of 13 animals from the treated group did not exhibit palpable tumor nodules on day nine after tumor transplantation. However, all mice in the control group had tumor nodules by this time. The complete remission (CR) achieved the maximal value of CR_max_ = 54% and lasted at a decreasing level of CR = 23% and 7% during the next 10 days (Table 3). In the remaining mice with palpable tumor nodules, significant and consistent remission value (TGI_max_ = 84%) was achieved by the 10th day after tumor transplantation.

**Figure 3:**
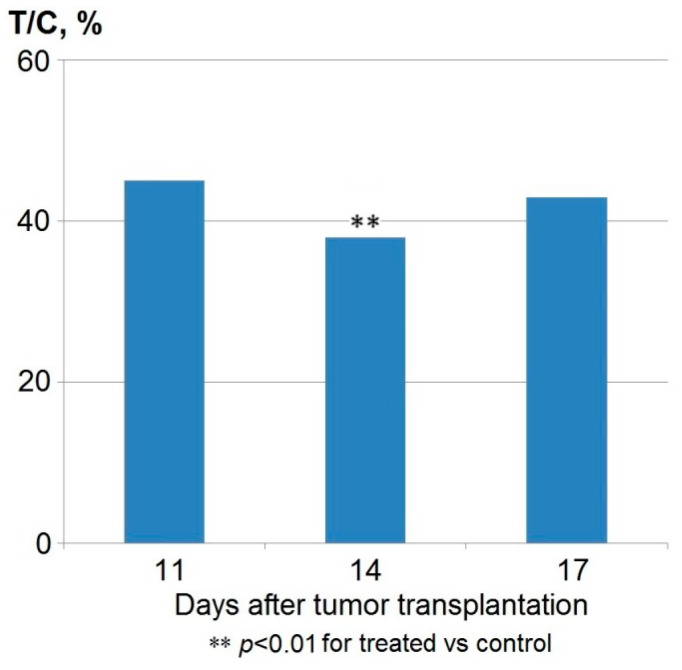
Tumor growth inhibition of human s.c. xenografts of breast cancer T47D after oral administration of anthrafuran (70 mg/kg for 5 days).

Finally, the investigation of anticancer activity of anthrafuran on xenografts of human triple negative T47D breast cancer was carried out using the optimal therapeutic schedule for the oral administration of anthrafuran which demonstrated the efficiency for treatment of the mammary adenocarcinoma Ca755 (70 mg/kg daily for 5 days, for 2–6 d after tumor transplantation). It was shown (Figure 3) that on the 14th day after tumor transplantation (seventh day after the treatment), T/C was 38% (*p* < 0.01) (minimum criterion T/C < 42%). The standard growth dynamics of T47D is characterized by exponential growth for 14 days (peak exponent) after transplantation, then, the growth curve quickly passes into the stationary phase, which is accompanied by a decrease in its sensitivity to cytotoxic therapy with anthrafuran. The negative dynamics of T/C in this experiment observed on the 17th day, show that multi-course treatment with anthrafuran is required to maintain the anticancer effect, which is typical for the overwhelming number of antitumor chemotherapies. Thus, to obtain a significant antitumor effect on xenografts of human breast cancer, a lower total dose of anthrafuran (ED_Σ_) was required than for the syngenic P388 and LCC tumors (350 mg/kg vs. 400–500 mg/kg).

### 2.2. Acute Toxicity

Table 4 shows the acute toxicity results for oral as compared with i.p. single administration of anthrafuran to the healthy mice. These data show that LD_50_ and LD_10_ values did not depend on gender but did depend strongly on the route of administration. The value of LD_50_ of anthrafuran administered orally was 6-times lower than that for i.p. administration (LD_50_ = 306.7 mg/kg vs. 52.5 mg/kg). The signs of acute toxicity caused by anthrafuran were also different for oral as compared with i.p. administration. After the i.p. injection of the highest doses, the animals in all groups died within 1–24 h with signs of cardiovascular failure caused by the neurotoxic effect of the drug. The oral administration of the highest doses of anthrafuran led to mice death within 3–5 d with symptoms of gastrointestinal toxicity and within 7–10 days due to hematological toxicity. The LD_50_ value for anthrafuran for oral administration corresponds to the third category of substance toxicity (~300 mg/kg) according to the Globally Harmonized Classification System [21].

## 3. Materials and Methods

### 3.1. Materials

Amorphous (*S*)-3-(3-aminopyrrolidine-1-carbonyl)-4,11-dihydroxy-2-methylanthra[2,3-*b*]furan-5,10-dione methane sulfonate dihydrate (anthrafuran, purity 99.4%) was synthesized as previously described [13]. The substance was dissolved in 0.2–0.5 mL of 5% glucose for i.p. treatment or in potable water for p.o. treatment at the necessary concentration to provide the needed dosage during 5 d of therapy or as a single dose for toxicity investigation. The solution was prepared by heating anthrafuran in a water bath to 90 °C for 5 min with light stirring. The treatment was performed 2–6 days after tumor transplantation. Mice in the control groups received solvent in the corresponding volumes and mode of application. The drug was administered to mice once a day in the appropriate individual doses using individual sterile plastic syringes.

### 3.2. In Vivo Tumor Models and Mice

The animal study was performed in accordance with the European Convention for the Protection of Vertebrate Animals, Directives 86/609/EEC [22], European Convention for humane methods for animal welfare and maintenance [23], the National Standard of the Russian Federation R 53434–2009 “Good Laboratory Practice” [24], and approved by the Ethics of Animal Experimentation of Gause Institute of New Antibiotics.

For the preclinical study, the following transplantable murine tumor strains were chosen: Ca755 mammary adenocarcinoma, i.p. P388 lympholeukemia, and s.c. LLC Lewis lung carcinoma (ATCC^®^ CRL-1642). For the final study, a human breast cancer s.c. xenograft of T47D with an autocrine regulation of cell proliferation by estrogen receptor-alpha (ATCC^®^ HTB-133) was used. All tumor strains were obtained from the Cryobank of the FSBI “National Medical Research Center of Oncology of N.N. Blokhin”, Ministry of Health of Russia (NMRCO) (Moscow, Russia).

Evaluation of antitumor activity was performed using BDF_1_ (C57Bl6j × DBA_2_) mice. To obtain the inoculating material, each murine strain tumor model was transplanted twice in DBA_2_ mice (*n* = 6–10) for P388, C57Bl6j male mice (*n* = 10–13) for LLC, and Ca755 or female Balb/c nude mice (*n* = 10) for T47D. Then, a suspension of 1 × 10^6^ leukemic cells or a 50 mg/0.2 mL suspension of tumor tissues in a culture medium 199 were prepared and inoculated i.p. or implanted s.c. into each mouse according to the type of tumor.

Inbred mice for the transplanted murine tumors were obtained from “Stolbovy” Nursery of laboratory animals (Russia) and maintained under standard laboratory conditions at the special Animal Department of the NMRCO. For the experiments with s.c. human tumor xenografts, we used thirty 8-week-old female Balb/c nude mice, weighing 20–22 g, from the virus-free vivarium at the NMRCO [25]. Animals were randomized into groups (*n* = 10). Mice in the control group received daily a saline solution following the schedule and doses given to the treated groups. The experimental evaluation of animals was carried out under the previously described conditions [26,27]. Therapy tolerance was evaluated on the basis of mice appearance and behavior such as general condition, behavior, attitude to food and water, and motor skills. For solid tumor models (LLC, Ca755, and T47D), the duration of the experiment limited by the maximal size of a tumor in the control group (≥2 cm^3^), after which the experiment was terminated. All animals in the control and in the treatment group were euthanized under general anesthesia using an ether overdose.

### 3.3. Evaluation of Antitumor Activity

To calculate treatment efficacy, we used standard criteria of survival and increasing of life span of T/C ≥ 125% for the mice transplanted i.p. with P388 (*n* = 6–10) and of tumor growth inhibition of TGI ≥ 50% for regular mice (*n* = 10–13), or T/C ≤ 42% for nude mice (*n* = 10) with s.c. tumors [26]. T/C and TGI (tumor growth inhibition) are standard criteria for evaluating the antitumor efficacy [26]. For P388 leukemia, model T/C was calculated as the ratio *L_Treat_/L_Control_* × 100% where *L_Treat_* and *L_Control_* are average life span in the treated and control groups, respectively. TGI was calculated as the ratio (*V_Contro_*_l_ − *V_Treat_*) / *V_Control_* × 100% where *V_Treat_* and *V_Control_* are average volumes of the tumor in the treated and control groups, respectively). The tumor volume was calculated as *V = (a × b × c),* where *a*, *b,* and *c* are the length, width, and height of the tumor nodule (mm). For the T47D model, ***T/C*** was calculated as the ratio *V_Treat_/V_Contol_* × 100% where *V_Treat_* and *V_Control_* are average tumor volumes in the respective cohorts. All abovementioned criteria of significant and reliable antitumor efficacy corresponded to the requirements mentioned elsewhere [23]. In addition, the criterion of complete remission (CR%, *n* = 13) was used for LLC, which was determined as the absence of the palpable tumor in drug treated mice. The autopsy of the dead mice revealed peritoneal leukemic carcinomatosis, an increase in the size of lymphatic nodules, and the presence of ascites fluid in the peritoneal cavity [28]. During therapeutic experiments, we determined the optimal range of treatment doses and observed treatment tolerability using standard conditions. The statistical analysis of the obtained data was performed with the Fisher’s exact test in Microsoft Office 2010 Excel. Significant differences were calculated for *p* < 0.05.

### 3.4. Acute Toxicity

Healthy BDF_1_ (C57Bl6j × DBA_2_) mice (18–20 g) were randomized into 20 groups (10 cohorts of males and 10 for females) (*n* = 6) and received anthrafuran in 5% glucose solution with single doses of 30, 40, 50, 60, and 70 mg/kg i.p. or 100, 200, 300, 400, and 500 mg/kg p.o. The acute toxicity was determined on the basis of mortality, survival time, and clinical manifestation of intoxication. The 50% lethal dose (*LD*_50_) and 10% lethal dose (*LD*_10_) values and confidence interval of *LD*_50_ were calculated using the Litchfield and Wilcoxon method with the StatPlus 2006 AnalystSoft StatPlus software.

## 4. Conclusions

In summary, the experimental evaluation on murine transplanted solid tumors or leukemia and human s.c. xenografts of breast cancer revealed the high antitumor potency of anthrafuran given orally. The anticancer effect of anthrafuran and the increasing life span of the mice with i.p. P388 leukemia with equivalent therapeutic doses and regimes of anthrafuran (five day schedule) were similar for both i.p. and oral administration, i.e., T/C_max_ = 214% (5 × 30 mg/kg [13]) vs. T/C_max_ = 219% (5 × 80 mg/kg p.o.), respectively. The signs of toxicity of orally administered anthrafuran were observed only for the single dose of 120 mg/kg. Moreover, orally administered anthrafuran for murine solid tumor models Ca755 and LLC showed a significant high long-term antitumor effect (TGI = 91% and 84%, respectively). Specifically, within two weeks after LLC tumor transplantation, several cases of complete remission (CR_max_ = 54%) were noted in the treated group. The antitumor activity of anthrafuran was also confirmed in human breast cancer xenografts implanted into nude mice. Thus, the optimal treatment schedule for orally administered anthrafuran includes single doses of 70–100 mg/kg for five days, which, in our experiments, led to a significant tumor growth inhibition or life prolongation for the different types of tumor models we used. An increase in the single effective dose for oral treatment correlates well with the results of the pharmacokinetics study for anthrafuran, which showed that the bioavailability of orally administered anthrafuran in rats reached 31% [18].

The evaluation of anthrafuran’s acute toxicity demonstrates that this agent is less toxic given orally than i.p. The ratio between the therapeutic and the toxic dose of the drug given orally was more than two times higher than for i.p administration. Overall, the results of our study on antitumor efficacy and acute toxicity of orally administered anthrafuran indicate the high potential for further development of this novel anticancer agent. Moreover, a highly significant antitumor effect was obtained with anthrafuran, which has a moderate level of bioavailability via oral administration. Therefore, the subsequent development of effective dosage forms that increase absorption of anthrafuran from the gastrointestinal tract can reduce single doses of anthrafuran and increase its effectiveness.

## Figures and Tables

**Table 1:** Life span of mice bearing P388 leukemia after oral administration of anthrafuran for a five-day course (5 × 24 h).

Group	Single Dose	Total Dose	Parameters	
			*L ± m* (days)	*T/C* (%)
Control	0.5 mL *	2.5 mL	9.5 ± 0.7	100
Anthrafuran	40 mg/kg	200 mg/kg	12.4 ± 0.9 **	130
	60 mg/kg	300 mg/kg	14.5 ± 1.1 **	153
	80 mg/kg	400 mg/kg	20.8 ± 3.2 **	219
	100 mg/kg	500 mg/kg	19.9 ± 3.7 **	202
	120 mg/kg	Lethal toxicity ***		

L ± m, mean lifespan with standard deviation and T/C, treatment-to-control ratio. * Water, ** significant differences between the control and treatment groups at *p* < 0.05 for all treated groups without differences between them, and *** single dose was toxic for this schedule.

**Table 2:** Oral administration efficacy of anthrafuran on i.p. P388 using different schedules.

Group	Single Dose	Regimen	Total Dose	Parameters	
				*L ± m* (days)	*T/C* (%)
Control	0.5 mL *	8 × 24 h	4.0 mL	9.7 ± 0.9	100
Anthrafuran	80 mg/kg	5 × 24 h	400 mg/kg	18.4 ± 1.7 **	190
		5 × 48 h	400 mg/kg	18.4 ± 2.1 **	190
		8 × 24 h	640 mg/kg	17.4 ± 2.2 **	179

L ± m, mean lifespan with standard deviation and T/C, treatment-to-control ratio. * Water and ** significant differences between the control and treatment groups at *p* < 0.05 for all treated groups without differences between them.

**Table 3:** Antitumor effect on s.c. Lewis lung carcinoma in mice (*n* = 13) after the oral anthrafuran treatment for 5 days.

Parameter	Days after Tumor Transplantation		
	9	13	19
TGI% *	84	61	49
Complete remission (number of mice)	7	3	1

* Tumor growth inhibition after exclusion of mice with complete remission.

**Table 4:** Acute toxicity of orally or i.p. administered anthrafuran in mice.

Route of Administration	Parameter	Doses, mg/kg	
		Males	Females
Intraperitoneal	*LD*_50_	52.5 (47.1 ÷ 57.9) *	53.1 (48.3 ÷ 58.4) *
	*LD*_10_	39.4	38.4
Oral	*LD*_50_	306.7 (209.1 ÷ 404.3) *	309.2 (237.4 ÷ 387.7) *
	*LD*_10_	128.7	129.9

LD_10_, 10% lethal dose and LD_50_, 50% lethal dose. * data are mean LD_50_ (confidence interval of LD_50_ for *p* ≤ 0.05).
